# Social inequalities in mental health and mortality among refugees and other immigrants to Sweden – epidemiological studies of register data

**DOI:** 10.3402/gha.v6i0.21059

**Published:** 2013-06-27

**Authors:** Anna-Clara Hollander

**Affiliations:** Division of Social Medicine, Department of Public Health Sciences, Karolinska Institutet, Stockholm, Sweden

**Keywords:** social inequalities, mental health, mortality, refugees, immigrants, register data

## Abstract

The aim of this PhD project was to increase knowledge, using population-based registers, of how pre- and post-migration factors and social determinants of health are associated with inequalities in poor mental health and mortality among refugees and other immigrants to Sweden. Study I and II had cross-sectional designs and used logistic regression analysis to study differences in poor mental health (measured with prescribed psychotropic drugs purchased) between refugee and non-refugee immigrants. In Study I, there was a significant difference in poor mental health between female refugees and non-refugees (OR=1.27; CI=1.15–1.40) when adjusted for socio-economic factors. In Study II, refugees of most origins had a higher likelihood of poor mental health than non-refugees of the same origin. Study III and IV had cohort designs and used Cox regression analysis. Study III analysed mortality rates among non-labour immigrants. Male refugees had higher relative risks of mortality from cardiovascular disease (HR=1.53; CI=1.04–2.24) and external causes (HR=1.59; CI=1.01–2.50) than male non-refugees did, adjusted for socio-economic factors. Study IV included the population with a strong connection to the labour market in 1999 to analyse the relative risk of hospitalisation due to depressive disorder following unemployment. The lowest relative risk was found among employed Swedish-born men and the highest among foreign-born females who lost employment during follow-up (HR=3.47; CI=3.02–3.98). Immigrants, and particularly refugees, have poorer mental health than native Swedes. Refugee men have a higher relative mortality risk for cardiovascular disease and external causes of death than do non-refugees. The relative risk of hospitalisation due to depressive disorder following unemployment was highest among immigrant women. To promote mental health and reduce mortality among immigrants, it is important to consider pre- and post-migration factors and the general social determinants of health.

Socio-economic conditions, living and working conditions, and social networks are among the social determinants that influence health (1). If the social determinants of health are unevenly distributed such as worse living conditions for one group or less healthy lifestyles for another, this may create social inequalities in health. Numerous studies have described the positive association between socio-economic status and health and longevity – the social gradient (1).

From the earliest studies in psychiatric epidemiology until today, psychiatric illness has also been found to be positively associated with social adversity (2). For depressive disorders, causation often explains the disorder, but for schizophrenia, the general explanation is selection (2). However, both for depressive disorder and schizophrenia, genetic vulnerability also plays an important role.

Immigrant-specific social determinants of health are often separated into pre- and post-migration factors (3). The association between social determinants of health, pre- and post-migration factors, and poor mental health and mortality are not same for all. Different factors, such as ethnicity and gender and other factors, modify the association.

Iyer et al. state, ‘It has gradually been recognised that different axes of social power relations, such as gender, socioeconomic position, discrimination and racism, are interrelated, not as additive but as intersecting processes’ (4). Llácer and colleagues highlight the lack of studies that link gender, migration, and health empirically, and they address the need to integrate a gender perspective into epidemiological studies on migration and health (5).

The four most commonly discussed pathways between the unequal distributions of social determinants of health and health inequalities are material deprivation, psychosocial factors, health behaviours, and access to health and social care (6). In addition to the four commonly discussed pathways, studies focussing on refugee mental health often add trauma as part of the psychosocial factors (7).

The frameworks of social determinants of health rarely include immigrant-specific determinants of health, except ethnicity (for an exposé of different frameworks of social determinants of health, see Ref. 6). Both Malmusi et al. (8) and Ingleby (9) stress that migration and health issues need to be integrated within the health equity framework. Ingleby suggested an intersectional perspective in migration and health issues in order to include it in the health equity framework (9).

## Previous empirical studies

In terms of pre-migration factors, studies have found that both income level in the immigrant's country of origin (10) and the reason for immigration (such as the need for asylum, work, or family reunion) (11) are associated with poor mental health among immigrants. Labour immigrants have a lower prevalence of poor mental health than refugees do (11).

There are many post-migration factors that seem to be of importance, such as the amount of time in the new country (12); social networks; language; acculturation status; socio-economic position in the new country (13); status loss (12); labour-market attachment (13); and experience of discrimination and racism (14).

There are specific post-migration factors for refugees. Long asylum procedures (15), arrival before family members, and worries of family left back home (16) restricted working opportunities, higher age, and living in institutions (17) are all associated with poor mental health. Bogic et al. found that the war-related factors were related to Post-traumatic Stress Disorder (PTSD) and that post-migration factors were related to the rates of mood, anxiety, and substance abuse disorders (18).

The hypothesis that immigrants have a higher prevalence of schizophrenia and psychosis than natives was proposed, tested, and supported as early as 1932 (19). These findings have been reproduced many times since (20). Common mental disorders refer to depressive disorders, anxiety disorders, obsessive–compulsive disorders and phobias. Studies have found that immigrants to the United States have lower levels of common mental disorders compared with natives, although this level seems to vary by country of origin and age at immigration (21). These lower levels compared with natives do not seem as apparent in Europe (22), although country-specific studies show conflicting results (23). Some researchers hypothesise that migration would be a risk factor for suicide, although many studies have found this it is still not firmly confirmed, according to Portzky et al. (24). There are ethnic differences in the risk of suicide in Sweden (25) and elsewhere (26).

Immigrants from low-income to high-income countries have, on average, a lower socio-economic position than the native population (27). Still, immigrants often display a lower mortality (28–30) than both the native population and their compatriots back home (31, 32). One explanation for the superior health could be that high-income countries accept labour migrants because there is a need for them in the labour force. Being in the labour force requires good health. This selection hypothesis is coined ‘The Healthy Migrant Effect’. Immigrants’ health deteriorates more rapidly with age than natives’ health does with age (8). There are also ethnic differences in mortality patterns (28–30). A range of studies has shown that certain kinds of stress are associated with cardiovascular mortality: both acute stress, such as combat stress (33) and stress after earthquakes (34), as well as chronic stress, such as marital problems (35) and low-control, high-demand work situations (36).

Ethnicity is difficult to define in general, and particularly as a variable in an empirical study in epidemiology. Perceptions and expressions of mental health and the stigma surrounding it differ across cultures and contexts (13, 37). Still, there is strong support for the idea that although poor mental health can have many expressions, it exists in similar forms cross-culturally (37, 38). In addition, there are differences in psychiatric health services utilisation between immigrants and natives (39).

Gender differences in health are neither just biological nor just socially constructed (40). The International Organisation for Migration describes how women are often seen as passive immigrants, whereas men are depicted as active, and how this representation colours perceptions of immigrants’ needs (41). Female immigrants from countries in conflict are often assumed to be members of the refugee's family (42), despite the fact that equal shares of the world's refugees are women.

Questions remain of how pre- and post-migration factors and social determinants of health are associated with inequalities in poor mental health and mortality among immigrants (9). Few previous studies have compared refugees with non-refugees of the same origin who immigrated to the same country at the same time. Thus, there is a question whether the higher likelihood of poor mental health among immigrants from low-income countries to high-income countries could be attributed to refugee-specific pre-migration factors and whether there is an interaction between origin and poor mental health among refugees. Little is known about whether refugees to high-income countries have higher mortality than non-refugees have. In addition, there is a knowledge gap in how the combination of gender, being foreign-born, and unemployment is associated with poor mental health.

The overarching aim of the PhD thesis was to increase knowledge of how pre- and post-migration factors and social determinants of health are associated with inequalities in poor mental health (measured by prescribed and purchased psychotropic drugs or hospitalisation for depressive disorder) and mortality among refugees and other immigrants to Sweden.

### Research questions

1. Are there differences in poor mental health (as measured by prescribed and purchased psychotropic drugs) between refugees and non-refugee immigrants, and could the hypothesised differences explain mental health differences by country or area of origin?
2. Do refugee immigrants have higher mortality rates than non-refugee immigrants?
3. Does the combination of general social determinants of health and post-migration factors increase inequalities among men and women in the relative risk of hospitalisation due to depressive disorder?
4. Are there gender differences in how pre- and post-migration factors and social determinants of health are associated with poor mental health among immigrants?

## Materials and methods

These research questions were answered by four sub-studies. The sub-studies and their relationship to the research questions can be seen in Table 1.

**Table 1:** The relationships between the research questions and sub-studies

Research question	Sub-study	Reference
1, 4	I	57
1	II	58
2	III	59
3, 4	IV	60

### Population

In this PhD thesis, the definition of an immigrant is one who is born abroad, but settles in Sweden, according to the Swedish bureau of official statistics, Statistics Sweden (43). This definition excludes children of immigrants born in Sweden (so-called second-generation immigrants), asylum seekers, and undocumented immigrants.

Sweden has a limited history of colonising other nations compared with other European countries (44) and was never drawn into World War II. During the final years of the war, Sweden admitted Scandinavian and European refugees (45). From the late 1940s and into the beginning of the 1970s, immigration to Sweden was characterised by labour migration (46). In the late 1960s, labour migration became more restricted. These restrictions did not apply to Nordic citizens, refugees or relatives of earlier immigrants (47). In 1994, Sweden signed the European Economic Area (EES) agreement. Since the ratification of the EES agreement, the number of labour migrants from EES countries has increased steadily (43). The approval rate of asylum applications has gone down significantly. Of those applying in 1989, 80% were granted asylum. In 2002, the proportion was 17%, and in 2012 the proportion was 30%.

As a means of identification, all Swedish citizens or people living in Sweden with a permanent residence permit are assigned a personal identity number in the Population Registration System (43). After ethical approval and permission, researchers are able to link to registers for research purposes with the help of the personal identity numbers; the data are made anonymous after linkage. To be included in the studies, a person had to be 18–64 years of age and fulfil the criteria stated in Table 2, according to the Swedish Population Registration system (43). The number of persons in Sweden fulfilling the inclusion criteria at the time of the study determined the study sizes (see Table 2).

**Table 2:** The inclusion criteria and the total population, percentage women, and percentage refugees in the four studies

Study	Inclusion criteria/study population	Total population	Women (%)	Refugees (%)
I	All immigrants from Afghanistan, Iraq, Iran, the Middle East, Somalia, and the former Yugoslavia in 2006 who were granted a resident permit fewer than 10 years ago either for being a refugee or for the reason of family reunion with a refugee.	43,168	48.5	56.5
II	Part 1: All registered immigrants compared with all Swedish-born in the year 2006.	5,507,262	49.3	1.6
	Part 2: Immigrants from non-OECD-countries in 2006 who arrived in Sweden since 1993.	298,641	51.5	15.4
III	Non-labour-market immigrants (including refugees and non-refugees such as persons admitted for family reunion with a refugee and for humanitarian reasons) to Sweden in 1998–2006 who immigrated between 1992 and 1998.	86,395	49.3	24.2
IV	The total population in 2000–2006 with a strong connection to the labour market in 1999.	3,284,896	47.5	<0.5 (excluded)

In Study I, II, and III, persons who could be assumed to have left the country without informing Swedish tax authorities were excluded by methods described by Weitoft (48). There were several exclusion criteria in Study IV. Prior to the start of follow-up, all participants were followed during a washout period for 3 years, 1997–1999. Participants who were hospitalised for depressive disorders during the washout period were excluded from the cohort. Immigrants who obtained resident permits after the start of the washout period in 1997 were excluded. Refugees were excluded because studies have found them to be at a higher risk of poorer mental health than other immigrants (11). In study IV, there was censoring of persons who had left Sweden, from the year they left Sweden. Persons who died during the time of the study were censored from the year of death. All who were hospitalised due to a depressive disorder prior to being unemployed were censored throughout the study. Persons who were sick-listed for more than two-thirds of a year, were on a disability pension, or taking parental leave were censored from the year they left the labour force. Censoring the sick-listed was done to ensure that those who lost their jobs did not have a depressive disorder prior to transition to unemployment.

### Variables

Register-based studies use data in official registers collected for generic purposes. The exposure variables and covariates are outlined in Table 3. All exposure variables and covariates were retrieved from Statistics Sweden's Longitudinal integration database for health insurance and labour-market studies (LISA by Swedish acronym) and Statistic Sweden's Longitudinal database for studies of the immigrants’ integration (STATIV by Swedish acronym) (43). All studies were adjusted for age, education, and marital status. Swedish registers do not record race or ethnicity for ethical reasons but do register the immigrant's country of origin. Country or area of origin is sometimes used as a proxy for ethnicity. In the studies, immigrants’ origin was classified according to different principles (Table 3). In Study I, II, and III, exposure was the reason for immigration being defined as refugee or non-refugee. The definition of a refugee is according to the Swedish Migration Board (SMB) classifications and for the non-refugees group, these were defined differently in the different studies (Table 4).

**Table 3:** Exposure, covariates and outcome in Study I and II

Study	Exposure	Covariates	Outcome
I	*Reason for immigration* 1	*Country or area of origin*: Afghanistan, Iraq, Iran, the Middle East, Somalia, and the former Yugoslavia *Time in Sweden*	*Prescribed psychotropic drugs* from The Prescribed Drug Register
II part 1	*Reason for immigration* 1 *Origin*: Swedish-born^2^, from an OECD country or from a non-OECD country		
II part2 the non-OECD sub-set	*Reason for immigration* 1 *Origin*: Asia, Iraq^2^, Iran, Middle East, North Africa, Latin America, the former Yugoslavia, the former Soviet Union, and Sub-Saharan Africa	*Residence in Sweden*: Metropolitan area (Stockholm, Gothenburg, Malmö), or other *Time in Sweden* *Children at home*	
III	*Reason for immigration* 1	*Residence in Sweden*: Metropolitan area (Stockholm, Gothenburg, Malmö), or other *Time in Sweden* *Economic activity* measured as employment status	*Causes of death* according to ICD-9 and ICD-10 including all-cause mortality, neoplasms, cardiovascular disease, external causes or all other causes, from The Cause of Death Register
IV	*Unemployment*: Employed^2^ compared those who lost their employment *Combination*: Employed male Swedish-born^2^ compared with employed male foreign-born, unemployed male Swedish-born, unemployed male foreign-born, employed female Swedish-born, employed female foreign-born, unemployed female Swedish-born, and unemployed female foreign-born	*Economic resources* were measured by the gross individual median from paid employment together with all benefits based on social insurance	*Hospital admission for a depressive episode* defined as F32 by ICD-10 excluding recurrent depressive episodes and bipolar disorders, from The Hospital Discharge Register

1 See Table 4.

2 Reference category.

**Table 4:** The definitions of reasons for immigration (refugees and non-refugees)

Refugees		Non-refugee	
	
Reason for residence permit	Criteria	Reason for residence permit	Criteria
Refugee status/subsidiary protection status	Have reason to fear persecution in their native country due to race, nationality, religious or political beliefs, gender, sexual orientation, or membership in a particular social group.	Humanitarian reasons (Study II and III)	Circumstances in the immigrant's current life situation.		
Status as a person otherwise in need of protection	Have a well-grounded fear of suffering the death penalty or torture, or need protection due to internal or external armed conflict or environmental disaster in their native country.	Family of refugees (Study I, II and III)	Family of refugees, such as partners/spouses and children.		
Quota refugees are selected by either of the above reasons		Others (Study II)	All other immigrants.		

All outcome variables were taken from registers administered by the National Board of Health and Welfare, the specific registers in terms of outcome are specified in Table 3, under the heading Outcome. In Study I and II, prescribed psychotropic drugs were used as a proxy for poor mental health. To have purchased prescribed psychotropic drugs implies that a physician has clinically assessed the patient's symptoms as psychiatric in nature, and by filling the prescription the patient has confirmed the physician's decision. While using psychotropic drug purchases as a proxy is not ideal, other options also have drawbacks. One advantage of using psychotropic drug purchases as a proxy is that these include prescriptions to outpatients from psychiatry as well as from other medical disciplines, including general practice, the most common form of care. Immigrants, particularly refugees (49) to Sweden are more likely to use psychotropic drugs than those who are Swedish-born (50). The greater use of antidepressants is almost entirely accounted for by higher morbidity (50). For sedatives and hypnotics, the difference seems to have more to do with a difference in the treatment of minor psychiatric disorders between ethnic minorities in Sweden and Swedish-born residents (50). Prescribed drugs in Sweden are covered by the universal health insurance (51). The patients paid, on average, about 22% of the actual cost of the prescribed drugs (51). Attitudes among patients and prescribers and communication barriers influence interpretation of a patient's poor mental health (52). Consequently, some groups might have been prescribed lower and weaker doses. For this reason, the outcome variable was coded into a binary variable (has/has not been prescribed and purchased these psychotropic drugs).

In Study III, the outcome was mortality. For detailed causes, see Table 3. Statistics Sweden studied potential unregistered deaths and found a problem among immigrants older than 85 years of age (43). We addressed the risk of unregistered deaths by including only persons aged 18–64. Differences in unregistered deaths abroad by origin were addressed by adjusting for origin.

Study IV used hospitalisation due to a depressive disorder as an outcome. For a definition of Hospital admission for a depressive episode, see Table 3. The validity of the diagnoses in the register has been tested and was found to have an overall high quality (53). Swedish citizens and persons with a permanent residence permit pay 80 SEK (∼US Dollar 12), per night in hospital.

### Methods

Study I and II had a cross-sectional design. Study III and IV had a cohort design with time defined in years. In Study III, the follow-up started on 1 January 1998 whereas in Study IV it started in 2000. Both ended by censoring on 31 December 2006. The analyses were conducted with the SAS software package 9.2. Because of large gender differences in the prevalence of the health outcomes for men and women, all four studies presented results for men and women separately. In addition to the gender-stratified analyses in Study IV, results were also presented with gender as a covariate.

In Study I and II, demographic variables were analysed using Chi-square tests. The association between the exposure, covariates, and the outcome of psychotropic drugs purchased was analysed using logistic regression. The −2Log Likelihood value was used to assess what model had the best fit. Results were presented as odds ratios (OR) with 95% confidence intervals (CI_95_).

In Study III, the incidence rates were calculated as deaths per 10,000 person-years. Demographic variables were compared using Chi-square and *t*-tests. Cox regression models were used to estimate hazard risk ratios (HR) for all-cause mortality and cause-specific mortality. These models were adjusted for age, age and origin, and age, origin, and covariate variables, respectively. The explanatory variables included in the final model were selected in a stepwise process. Tests of statistical power, as well as graphical and statistical tests of fulfilment of the Cox regression assumption of proportional hazards, were performed as suggested by Hosmer (54). Results were presented as HR with CI_95_.

For Study IV, incidence rates were calculated as the number of hospital episodes for a depressive disorder per 10,000 person-years. Cox regression models were used to estimate HR. The transition from employment to unemployment was coded binary, and subjects were split into an exposed and a non-exposed group and treated as distinct groups throughout, as suggested by Clayton and Hills (55). Five models were fitted. Best fit was tested with a stepwise procedure. Tests of statistical power, as well as graphical and statistical tests of fulfilment of the Cox regression assumption of proportional hazards, were performed as suggested by Allison (56). Results were presented as HR with CI_95_.

### Ethical considerations

All studies were approved by Stockholm Regional Ethical Review Board (2008/732-31).

## Results

Study I showed that refugees, both men and women were significantly more likely to have poor mental health (as measured by prescribed and purchased psychotropic drugs) (for men OR=1.47; CI_95_=1.32–1.64, for women OR=1.53; CI_95_=1.41–1.66) than the referent non-refugees category. When adjusting for all covariates, refugee women in comparison to non-refugee women were found to have a significantly higher likelihood of poor mental health (OR=1.27; CI_95_=1.15–1.40); non-refugee men, however, did not differ significantly from refugee men (OR=1.07; CI_95_=0.95–1.20).

Study II showed that among non-OECD immigrants most refugees, but not all, had a higher likelihood than non-refugees. Compared with non-refugees, who were the referent category in each strata, refugees from Asia (men OR=1.40; CI_95_=1.19–1.65, women 1.96; CI_95_=1.69–2.28), Iraq (men OR=1.14; CI_95_=1.04–1.25, women OR=1.32; CI_95_=1.20–1.46), the Middle East (men OR=1.46; CI_95_=1.21–1.76, women OR=1.49; CI_95_=1.17–1.89), the former Yugoslavia (men OR=1.14; CI_95_=1.05–1.25, women OR=1.13; CI_95_=1.04–1.21), the former Soviet Union (men OR=1.47; CI_95_=1.08–1.99, women OR=1.37, CI_95_=1.07–1.75), as well as male refugees from Latin America (OR=1.27; CI_95_=1.03–1.56) had significantly higher likelihoods than non-refugees with the same origin. For immigrants from Iran, North Africa, and sub-Saharan Africa and female immigrants from Latin America, there were no differences between refugees and non-refugees.

In Study III, the unadjusted incidence rates differed between male and female refugees and non-refugee immigrants for all causes and specific causes. The statistical power was sufficient for all-cause mortality but weaker for some specific causes of death, especially cardiovascular disease and external causes for women. In the first model, adjusted for age, the relative risk of mortality did not differ between refugees and non-refugee immigrants. Neither did the risk differ for all-cause mortality nor for any of the specific causes, apart from a lower risk of neoplasm among refugees (relative risk of mortality for refugees HR=0.70; CI_95_=0.52–0.95). Adding country of origin to the model resulted in enhanced differences in the hazard ratios between male non-refugee and refugee immigrants for cardiovascular mortality (HR=1.58; CI_95_=1.08–2.33). Adding other covariates increased the hazard ratios for refugees compared with non-refugees for cardiovascular mortality among both women (HR=1.49; CI_95_= 0.86–2.59, not significant) and men (HR=1.53; CI_95_=1.04–2.24) and of external causes among men (HR=1.59; CI_95_=1.01–2.50). The results regarding cardiovascular mortality appeared to be negatively confounded by origin.

In Study IV, the unadjusted incidence rates indicated differences in rates of hospitalisation because of a depressive disorder by employment status during follow-up, by gender, and by immigrant status. The Cox-regression model with the best fit included transition to unemployment, gender, immigrant status, education, age group, and marital status (full model). The model that included only employment status and age group (first model) was compared with the full model. In the first model, persons who experienced unemployment had a relative over-risk of hospitalisation (HR=2.03; CI_95_=1.98–2.15) compared with those who did not experience it (reference category). Also in the full model, those who experienced unemployment had a higher relative risk (HR=1.94; CI_95_=1.85–2.03) compared to the employed. In the full model, being female, being foreign-born, having low education, and not being married increased the relative risk for hospitalisation due to depressive disorders. To test if the combination of transition to unemployment, being female, and foreign-born increased the relative risk of hospitalisation for depressive disorders, a model was created with the combined variable *employment status–gender–immigrant status*, adjusted for age group, marital status, and education. Employed Swedish-born men had the lowest relative risk of hospitalisation for depressive disorders. A higher relative risk but not significantly different from each other were employed male foreign-born (HR=1.31; CI_95_=1.17–1.46) and employed female Swedish-born (HR=1.50; CI_95_=1.43–1.58). Even higher but not significantly different from each other were employed female foreign-born (HR=2.14; CI_95_=1.94–2.37), unemployed male Swedish-born (HR=2.26; CI_95_=2.12–2.42), and unemployed male foreign-born (HR=2.45; CI_95_=2.13–2.82). Unemployed female Swedish-born had an even higher relative risk (HR=2.62; CI_95_=2.45–2.80), however, and unemployed foreign-born females had the highest relative risk (HR=3.47; CI_95_=3.02–3.98).

## Discussion

### Main findings

- There was a significant difference in poor mental health (as measured by prescribed and purchased psychotropic drugs) between refugees and non-refugees for most groups.
- Male refugees had a higher relative risk of mortality from cardiovascular diseases and external causes than male non-refugees.
- Employment status, gender, immigrant status, education, and marital status were all associated with the relative risk of hospitalisation due to a depressive disorder. Unemployed foreign-born females had the highest relative risk.

These results show that there are differences in poor mental health (as measured by prescribed and purchased psychotropic drugs) between refugees and non-refugee immigrants, and that it, in part, can explain mental health differences by country or area of origin. A study by Tinghög et al. found that socio-economic disadvantages explained low subjective well-being for European immigrants, but not for non-European immigrants (57). Study I and II adds to the Tinghög et al. findings that some of the poor mental health among non-OECD immigrants could probably be explained by a higher likelihood of poor mental health among the refugees present in this group. Immigrants to the United States have been found to have common mental disorders on a par with natives in most cases (21). This is not apparent among immigrants in Europe (22). The high and varying proportions of refugees might be one of many reasons for the varying results in studies of common mental disorders among immigrants in Europe.

A number of interpretations could be proposed to explain why the reason for immigration had an impact in some groups but not in others. One is the interaction with the country of origin. Immigrants from countries or areas of origin with large differences between refugees and non-refugees might come from places where the conditions could make the consequences of persecutions worse. Immigrants from countries or areas of origin with little difference between refugees and non-refugees might originate from countries where the conditions are similarly bad for those who qualify for asylum and those who obtained residence permits for other reasons.

Another interpretation could be that asylum seekers from some countries or areas might be met with more suspicion than others. Besides being subjected to the threats of persecution in the country of origin, the former group also experiences a more hostile asylum process in the new country. According to this explanation, the differences are not in pre-migration factors but related to post-migration factors. Other post-migration factors could be differences in social networks, experiences of racism and discrimination, acculturation status, cultural integration, and status loss associated with the country or area of origin that modify the likelihood of poor mental health for refugees and non-refugees.

The finding that many refugees had a higher likelihood of poor mental health (as measured by prescribed and purchased psychotropic drugs) than their fellow compatriots underlines the previous criticism of attributing mental health differences to ethnicity or country of origin (58). The country of origin is a relevant variable when analysing the mental health of immigrants in epidemiological studies. Still, differences by country of origin or ethnicity can hide structural differences such as the reason for immigration.

Mortality among immigrants seems to be associated with pre-migration factors. This study shows that area or country of origin can confound mortality differences negatively, meaning that without adjusting for the country or area of origin, the differences between refugees and non-refugees were not visible. This shows that the country or area of origin is relevant in order to understand mortality differences. However, it does not reveal the whole picture.

What could explain the higher relative risk of cardiovascular mortality among refugees? Stress, including war combat injury (33), is known to be associated with cardiovascular mortality. One interpretation could be that exposure to refugee-specific pre- and post-migration factors are harmful in a manner similar to war combat injury. Another interpretation could be that the association between refugee-specific pre- and post-migration factors and mortality is mediated by lifestyle factors known to increase the risk of cardiovascular mortality. Depression and PTSD are independent risk factors for cardiovascular disease (59, 60). An additional interpretation of the results could be that poor mental health among refugees increases the relative risk of cardiovascular mortality.

For male refugees there was an increased relative risk of death from external causes. Suicide accounted for about 40% of the external causes of death among men. There was not enough statistical power to test the relative risk of suicides separately. Study I and II, and many other studies, show that refugees have a higher likelihood of poor mental health. Persons with poor mental health have a pronounced over-risk of suicide, and studies show that refugees have a higher risk of suicidal thoughts and attempts (61). Fortune stresses the need for more studies on suicides among refugees (62). As already stated, this could not be done in Study III. The findings of this study could be an indication that the ethnic differences in suicide rates mask pre-migration factors.

Only refugee men had significantly higher relative risks of mortality compared to non-refugee immigrants. This could be due to low statistical power among women. However, other explanations are possible. The results of Study I and III correspond to gender differences in Sweden, with a higher likelihood of poor mental health for women compared to men but lower mortality among women compared to men (63).

Llácer et al. highlighted the need for epidemiological studies on gender, migration, and health (5). The combination of the variables of unemployment, gender, and immigrant status makes the social determinants even more unevenly distributed, and this creates additional inequalities. Study IV shows that it is possible and feasible to use the intersectional perspective in epidemiological research when studying migration and health in an equity framework.

### Methodological considerations

Studies show a need for studies with large sample sizes when studying mental health in refugee populations in order to reduce the risk of type 1 errors (64, 65). The advantages with register studies are the large number of participants; disadvantages include the lack of control of cross-cultural issues, discrimination and racism and information concerning the social determinants of health in the country of origin that might be influential.

When conducting research on mental health using register data from health care sources, the researcher has to rely on physicians reporting accurate diagnoses. If immigrants are compared to natives, cross-cultural misdiagnosing could create information bias. In Study I and the second part of Study II, this bias was partly avoided by comparing immigrants with immigrants and adjusting for country or area of origin. In the first part of Study II and IV, immigrants were compared with natives. In Study IV, hospitalisation due to depressive disorders implies severe cases at psychiatric wards. Thus, specialist psychiatrists give the diagnoses. This should lower the risk of information bias.

One way of lowering the random and systematic errors due to cross-cultural mis-diagnosing is to avoid specific diagnoses. Instead, the outcome can be dichotomised into having poor mental health or not, such as in Study I and II. However, in the United Kingdom, South Asians have been found to be assigned mental health problem less often than white English despite having similar symptoms (52). This phenomenon could lower the prevalence ratio and potentially create systematic errors.

The outcome of poor mental health in Study I and II was studied using the proxy measure psychotropic drugs purchased because it implies that a physician has clinically assessed the patient's symptoms as psychiatric in nature, and by filling in the prescription, the patient has confirmed the physician's decision. This choice of outcome has its limitations. First, it is not validated against any gold standard and lacks diagnostic control. Second, people who are prescribed psychotropic drugs are likely to have symptoms that are more serious; hence, the measure is likely to miss those with minor symptoms.

The study design in Study I, II, and III, comparing immigrants with immigrants from the same area or country of origin with different reasons for immigration, is novel. There are advantages and disadvantages of using the SMB's classifications of reasons for immigration. Currently, Sweden is very restrictive in terms of granting asylum. The threshold to get asylum is high and requires adequate evidence; the refugee category is thus probably a valid category. However, the non-refugee category could include misclassified refugees. Refugees misclassified as family of refugees or refugees admitted for humanitarian reasons would weaken the association between the reason for immigration and the likelihood of poor mental health and mortality risk.

### Implications

In terms of health care for immigrants, Ingleby stated, ‘…only sustainable, structurally embedded changes in all parts of the health system are capable of delivering the improvements that are needed’ (9, p. 232). This PhD thesis shows that this is especially true for health care for refugees and implies that health care targeting refugees needs to be part of the general health care chain, permanent and evidence-based.

The finding underlines the need for caution when interpreting results based on country of origin or ethnicity in epidemiological research. This does not imply that ethnicity or cultural practices are irrelevant in health care or in clinical settings. In fact, with a deeper understanding of cultural differences in the perception and expression of poor mental health, clinicians could improve not just health care but epidemiologic studies, also. Health care staff with a thorough understanding of cross-cultural differences will diagnose with greater accuracy; the diagnoses thus will be more valid. Valid diagnoses can in turn be used in register-based epidemiological research in order to find better ways to prevent poor health and promote public health.

Additional implications are that health care staff in all fields needs more education and training in the relevance of pre-and post-migration factors, ethnicity, gender, and social determinants for poor mental health and mortality among immigrants to in order to diagnose with more accuracy and improve health care for immigrants.

### Future studies

There is solid support for an immigrant's higher risk of schizophrenia and psychosis. No study has had the statistical power to test if refugees have an over-risk of schizophrenia and psychosis compared with non-refugees from the same countries. If refugees have an over-risk, this could strengthen the theory that social adversity has a role to play in the heightened risk among immigrant groups, and this will benefit a deeper understanding of the aetiology of schizophrenia.

## Conclusions

The studies supported the framework proposed in the background section, suggesting that pre- and post-migration factors and unevenly distributed social determinants of health create social inequalities in poor mental health and mortality among immigrants. The studies illustrate that in order to investigate poor mental health and mortality among immigrants, it is crucial to consider pre- and post-migration factors as well as general social determinants of health.
